# Landscape of paediatric endocrine clinical practice in Italy: results from a survey of the Italian Society for Paediatric Endocrinology and Diabetology (ISPED)

**DOI:** 10.1186/s13052-025-01940-w

**Published:** 2025-03-24

**Authors:** Maria Elisabeth Street, Anna Di Sessa, Andrea Esposito, Anastasia Ibba, Giorgia Pepe, Riccardo Bonfanti, Felice Citriniti, Giuseppe D’Annunzio, Maria Rosaria Licenziati, Malgorzata Wasniewska, Valentino Cherubini, Mariacarolina Salerno

**Affiliations:** 1 Department of Medicine and Surgery, University of Parma, Via Gramsci 14, Parma, 43126 Italy; 2 Unit of Pediatrics, University Hospital of Parma, Parma, Italy; 3 Department of Woman, Child, and General and Specialized Surgery, University of Campania “Luigi Vanvitelli”, Naples, Italy; 4 Department of Emergency, Santobono-Pausilipon Children’s Hospital, Naples, Italy; 5 Pediatric Endocrinology Unit and Newborn Screening Center, Pediatric Microcitemic Hospital, ASL Cagliari, Cagliari, Italy; 6 Department of Human Pathology of adulthood and childhood, University of Messina, Messina, Italy; 7 Pediatric Diabetology Unit, Department of Pediatrics, Diabetes Research Institute, IRCCS San Raffaele Scientific Institute, Vita Salute San Raffaele University, Milan, Italy; 8 Department of Pediatrics -Azienda Ospedaliero-Universitaria “R. Dulbecco”, Catanzaro, Italy; 9 Pediatric Clinic and Endocrinology, Regional Center for Pediatric Diabetes, IRCCS Istituto Giannina Gaslini, Genoa, Italy; 10 Neuro-endocrine Diseases and Obesity Unit, Department of Neurosciences, Santobono-Pausilipon Children’s Hospital, Naples, Italy; 11 Department of Women’s and Children’s Health, Azienda Ospedaliero-Universitaria delle Marche, Ospedali Riuniti di Ancona, “G. Salesi Hospital”, Ancona, Italy; 12 Department of Translational Medical Sciences, Paediatric Endocrinology Unit, University “Federico II”, Naples, Italy

**Keywords:** Pediatrics, Endocrinology, Pediatric endocrinology, Survey, Subspecialty, Shortage, ISPED, Clinical practice

## Abstract

**Background:**

Pediatric endocrinology has developed enormously over the last 30 years. Many conditions followed-up are rare and/or chronic complex diseases requiring a high level of expertise. Therefore, defining pediatric endocrinology workforce has become crucial. We aimed to provide an overview of the landscape of the Italian Pediatric Endocrinology centers.

**Methods:**

A national electronic survey on clinical endocrine practice among the Italian Society for Pediatric Endocrinology and Diabetes (ISPED) centers was carried out. The full time equivalent (FTE) was used to assess the time dedicated by healthcare providers (HCPs) to pediatric endocrinology and calculate the needs.

**Results:**

Ninety-one centers completed the electronic survey. Forty-four/91 centers had incorporated a pediatric diabetology service, while the remaining had an independent center. Among HCPs, 271 were pediatric endocrinologists (94 with a temporary, and 265 with a permanent contract). In 14/91 centers, adult endocrinologists were part of the medical staff. In 45/91 centers clinical activity was carried out five days a week. A mean FTE of 0.56 for medical doctors, 0.49 for nurses, 0.31 for dietitians, and 0.13 for psychologists was reported. An average of 110 patients with rare diseases was followed *per* centre *per* year. Based on the ISPAD international criteria for the FTE required for the care of diabetic youths we considered rare diseases as a reference instead of diabetes, without considering any other consultations, and this showed a shortage of 80% of required pediatric endocrinologists, 89% of needed nurses, 93% of required dietitians, and 94% of required psychologists. Moreover, approximately 20 pediatric endocrinologists were expected to retire within the following two years. Overall, a mean of 1148 consultations/year per centre was reported for each medical FTE (a mean of 367 first consultations, and 786 follow-ups). Education and training for growth hormone and other specific treatments were provided by a variety of HCPs, mainly by medical doctors (22/91 centers).

**Conclusions:**

At present pediatric endocrinology shows a significant burden of activity with a severe shortage of personnel. This should be addressed by policy makers in order to develop strategic programs to ensure optimal care. Recognizing pediatric endocrinology as a subspecialty and offering appropriate training programs would represent a significant step further.

**Supplementary Information:**

The online version contains supplementary material available at 10.1186/s13052-025-01940-w.

## Background

Pediatric endocrinology is a pediatric discipline that includes, and has as an essential part, auxology [1, 2]. Therefore, first of all, it is related to growth and comprises the study of both biological and endocrine factors, and second with puberty, sexual differentiation, and gonadal function [1]. Hence, pediatric endocrinology represents the backbone of any other pediatric subspecialty and the pediatricians involved are both case-managers of patients needing a multidisciplinary approach, and consultants for other disciplines [2]. Kiess et al. stated that “*Pediatric endocrinology is pediatrics. As pediatrics is always directly related to public health and the wellbeing of individuals and societies*,* pediatrics is public health*” [1]; indeed, pediatric endocrinology deals with the most common health risks as obesity, vitamin D deficiency, diabetes, thyroid diseases, short and high stature conditions, bone diseases, as well as with very rare and/or severe conditions as Prader-Willi, Noonan and Turner’s syndromes, adrenal disorders besides chronic inflammatory diseases, cystic fibrosis-related diabetes, consequences of organ transplantion and cancer treatments, and gender dysphoria, just to mention a few [1, 2]. Many of these conditions often have a limited visibility among advocacy groups which are sometimes missing, and this at variance other sub-specialties as neonatology, oncology and congenital heart diseases that have a much greater visibility and impact on institutions [1–4]. Finally, pediatric endocrinology plays an important role in all fields of research such as epidemiology, genetics and molecular biology, pathophysiology, drug discovery and treatments, and requires currently continuous and updated clinical practice guidelines, consensus, and position statements, and development of registries.

To date, in Italy, as well as in many other countries, from a clinical point of view the hub and spoke modality is the chosen approach to better serve the community. As per definition, it represents an organizational structure in which a central facility (the hub) offers the highest-level care with advanced and specialized care and services, and is connected to a number of smaller, local facilities (the spokes) typically providing more general or routine healthcare needs. This model optimizes service delivery by centralizing specialized care at a central “hub” while delegating routine services to smaller, local facilities or “spokes.” This approach enhances resource management, improves access to care, and leads to better patient outcomes.

The hubs should be required to have radiology, neuroradiology, nuclear medicine, surgery (pediatric, when available), and medical genetics units, biochemical and hormone laboratories, genetic laboratories. They must network with community pediatricians and specialists, general physicians, adult endocrinologists, cardiologists, ears-nose and throat specialists, ophtalmologists, dermatologists, onco-hematologists, pediatric wards, neonatologists, diabeticians, orthopaedics, physiatrists, neuropsychiatrists, dentists, etc. Adolescent gynecology should be considered also.

Moreover, the number of rare diseases is increasing for different reasons, with the need to make more molecular diagnosis for the identification along with new treatment opportunities.

Definitely, this pediatric subspecialty has developed enormously and increasingly over the last 30 years. Currently, pediatric training presents differences among European countries, and definitely on a worldwide basis [2, 5]. In Italy, sub-specialties are not recognized. The 5-year programme to train pediatricians at present considers short periods of time spent in elective sub-specialities [6] although it is well recognized by the European Society of Pediatric Endocrinology (ESPE) that a comprehensive basic training in pediatric endocrinology would require a minimum of 2 years [7, 8].

Despite its emerging role as subspecialty, there are no current national data on pediatric endocrinology activity in Italy. The Italian Society for Pediatric Endocrinology and Diabetes (ISPED) steering committee (2021–2023) prepared a survey to obtain the current landscape of pediatric endocrinology, and submitted it to its members, aiming at evaluating current pediatric endocrinology practices in Italy, to identify gaps, clinical and/or educational needs, future challenges, and areas for improvement. Finally, professional collaborations among pediatric endocrinologists are warranted to promote a cohesive approach to improving care standards nationwide.

## Methods

A nationwide survey was conducted from October to December 2022 among pediatric endocrinologists, members of the ISPED. This Society is a key organization for professionals in pediatric endocrinology and diabetes in Italy that plays a central role in scientific research, clinical practice, and overall care for children with endocrine and metabolic disorders. ISPED collaborates with other national and international scientific societies, organizations, and institutions with the aim to shape the future of pediatric endocrine care in Italy. The society is led by a steering committee, and organized into regional chapters that coordinate local activities. It includes specific national committees focused on key areas and needs for pediatric endocrinology.

ISPED members were asked to fill out an online 50-item questionnaire grouped into five sections to provide a comprehensive overview of the centers. We also asked that only one member/center should fill in the questionnaire, and we asked to report data referring to the year 2021. The first sections of the questionnaire were related to data specific to the Center, and to its staff. Information on patients and services was included in the last section of the questionnaire. An English version of the survey is presented as Supplement 1 (S1).

Demographic data on the Italian resident population by region were collected from the Italian National Institute of Statistics (ISTAT) registry [9].

To enhance participation and maximize the response rate, a standard procedure in terms of follow-up calls and email reminders was carried out. Data provided by the HCPs were collected on an online platform (survey monkey) and then exported for statistical analysis as an excel file. Any questions needing further details were completed using follow-up calls.

We used the full-time equivalent (FTE) [10, 11] to measure the effective time dedicated by HCPs to pediatric endocrinology. An FTE of 1.0 is equivalent to a full-time worker. The actual FTE was calculated based on the number of official hours dedicated per week to pediatric endocrinology activities and considering 38 h/week as full time. To calculate any shortage of personnel within the health care system, we applied the criteria set for patients with diabetes by ISPAD [12], as most rare diseases have a similar workload to diabetes, and there is a general lack of criteria that can be applied for paediatric endocrinology for the Italian reality [13]. Furthermore, it is crucial to consider that each pediatric endocrinologist manages a large number of other patients other than rare diseases that have not been considered in this workload, thus, calculations are certainly underestimated.

The questionnaire was focused on measurable and officially recognized activities, thus on “Front Office” activities. None of the “back-office” activities were taken into consideration.

### Statistics

Descriptive analyses were used to describe the main features of the Centers, staff, and patients. Differences between categorical variables were analyzed using the Chi-square test or Fisher’s exact test. The normality of data distribution was assessed with the One-sample Kolmogorov–Smirnov test. For non-normally distributed data, variables were log-transformed prior to analysis. Continuous variables were summarized as means ± standard deviations (SD) or medians ± interquartile range (IQR), as appropriate, while categorical variables were presented as frequencies and percentages.

The IBM SPSS Statistics software, Version 24 (IBM, Armonk, NY) was used for all analyses.

## Results

### Pediatric endocrinology centers

**Fig. 1:**
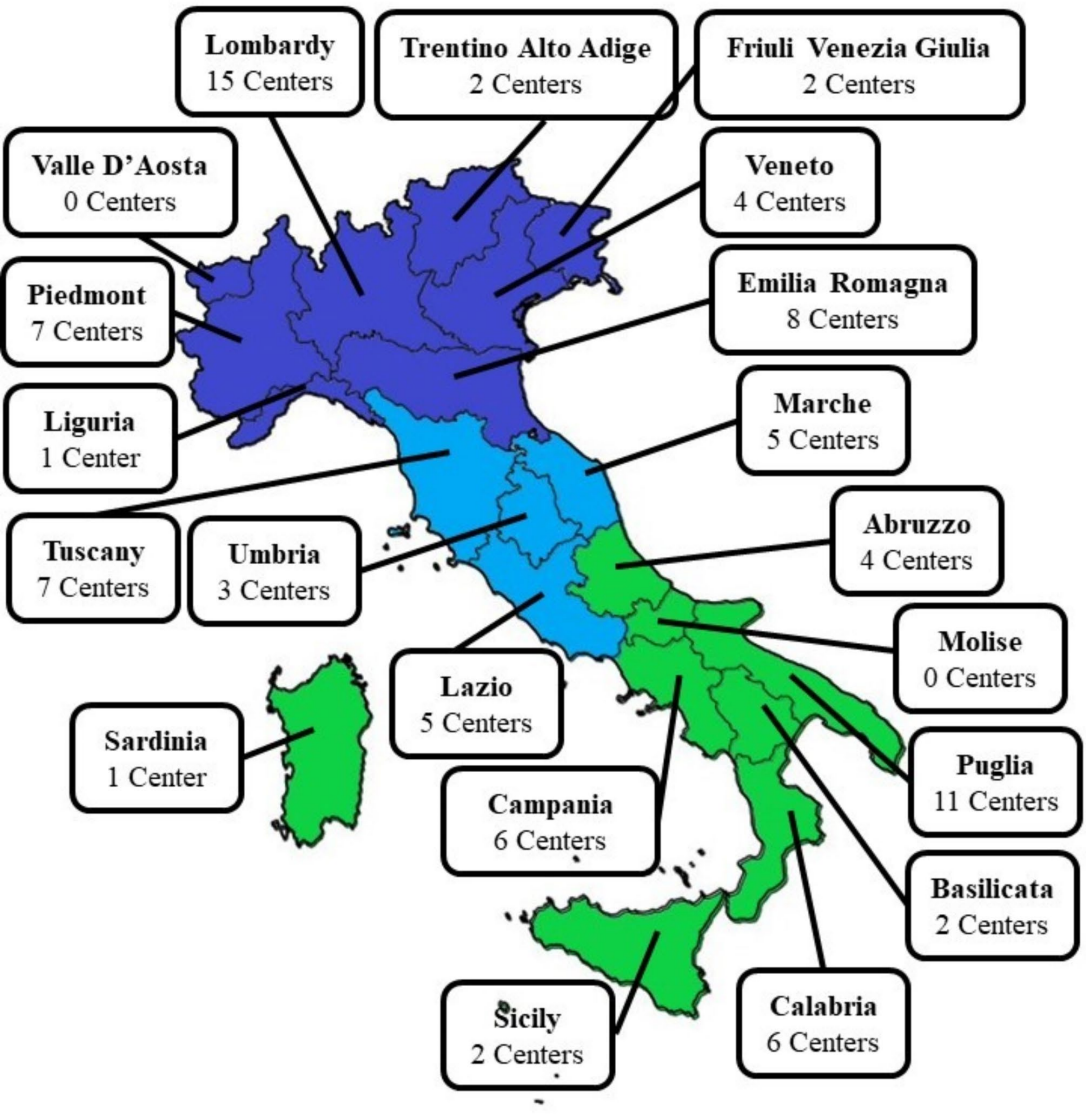
Regional distribution of ISPED pediatric endocrinology centers in Italy. Response rate:100%, no missing data

A total of ninety-one centers registered and completed the survey. The distribution throughout Italy is represented in Fig. 1. 39/91 centers were localized in Northern Italy, 20 in the Center, and 32 in Southern Italy. Distribution was not uniform and always corresponding to the residing population.

Sixty-nine out of 91 centers (75.8%) were hospital units, whereas 20 (22%) were academic centers embedded in teaching hospitals; the remaining 2 centers (2.2%) were clinics in local community health facilities. Sixty-one out of 91 centers (67%) were clinics within paediatric units, 14 (15.3%) were divisions within units, 6 divisions (6.6%) were within departments, and 6 (6.6%) were totally independent units.

**Fig. 2:**
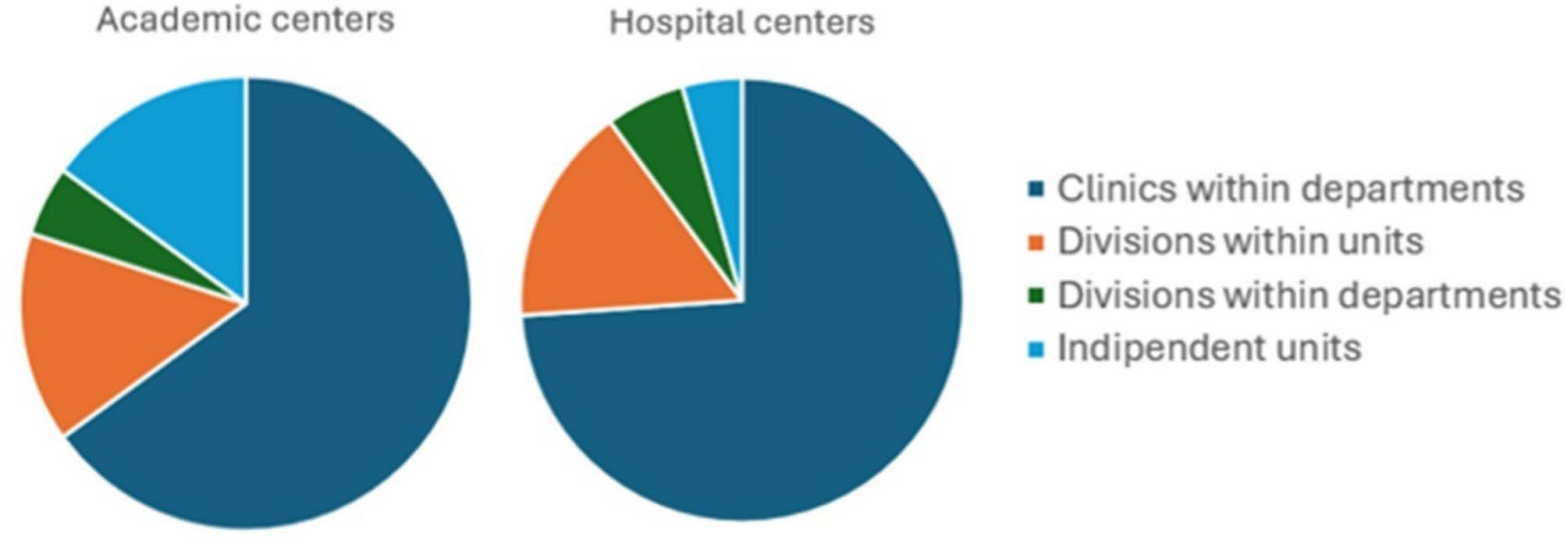
Main features of the ISPED pediatric endocrinology centers

More specifically, among 69 hospital centers, 51 were clinics within paediatric units, 11 were divisions within units, 4 were divisions within departments, and 3 were totally independent units. Among 20 academic centers, 13 were clinics within paediatric units, 3 were divisions within units, 1 was a division within departments, and 3 were totally independent units (Fig. 2).

Forty-four out of 91 centres (48.3%) centers had incorporated a pediatric diabetology service. The remaining centers had an independent pediatric diabetology center.

### Clinical activity

About half of the centers (45/91) carried out clinical activities five days a week, 2 centers less than once a week (a small clinic), and 6 centers 6 days per week. By examining the remaining centers, 11 worked once a week, 16 twice a week, 10 three times a week, and 1 four times a week.

In few centers (4/91) no telephone service was available for patients to contact the center, whereas 27 centers provided a landline telephone service at a set time, 17 had medical availability by landline or mobile telephone five days a week, and 43 centers provided medical availability by landline or mobile telephone 24 h as an unofficial service all week.

### Patient distribution

**Fig. 3:**
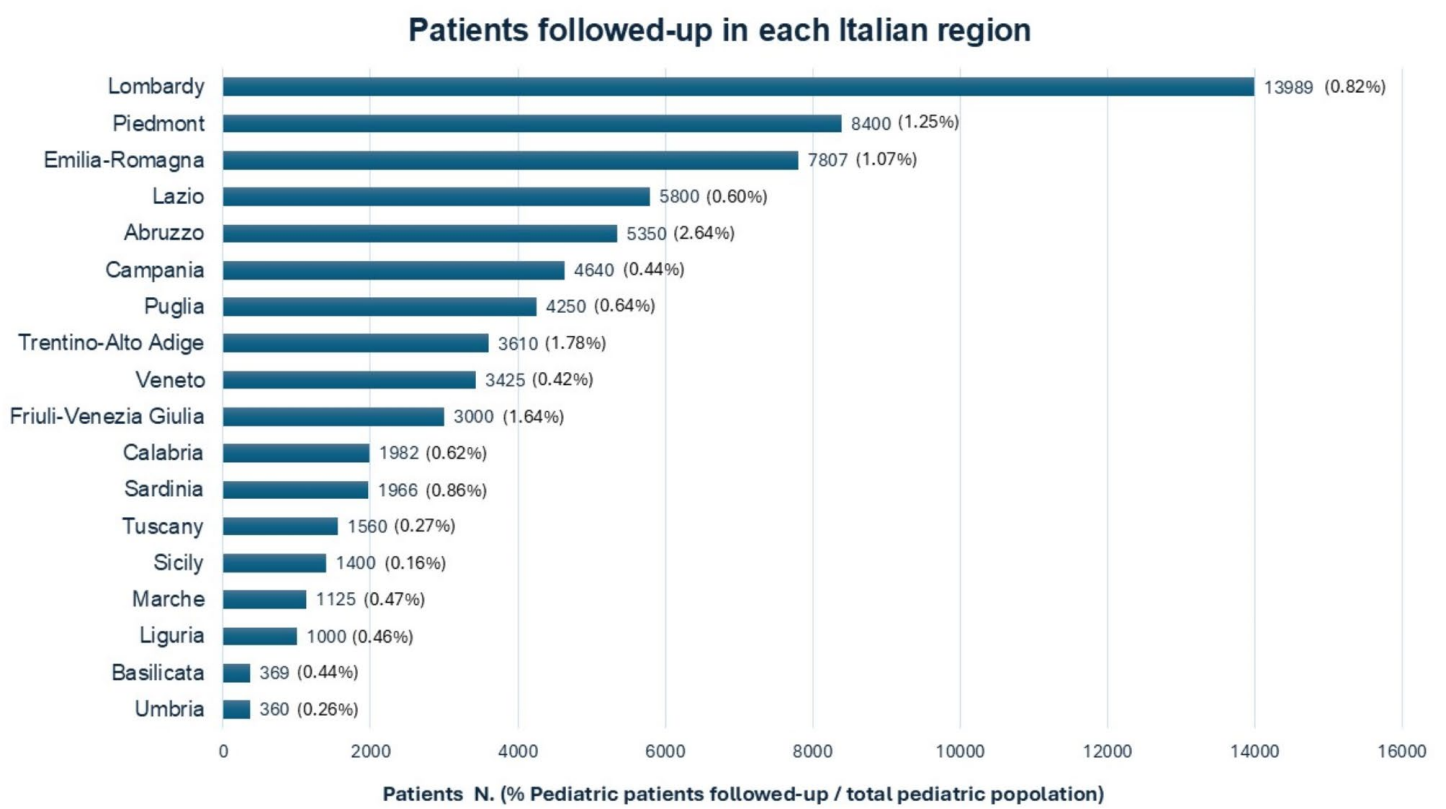
Distribution of patients followed-up in 91 ISPED pediatric endocrinology centers in 18 Italian regions (missing data: 2/91 centers). The percentage of pediatric patients followed-up with respect to the total pediatric population (0–18 years) per region is reported in brackets

The total number of patients followed-up in each region in 2021 is reported in Fig. 3.

**Fig. 4:**
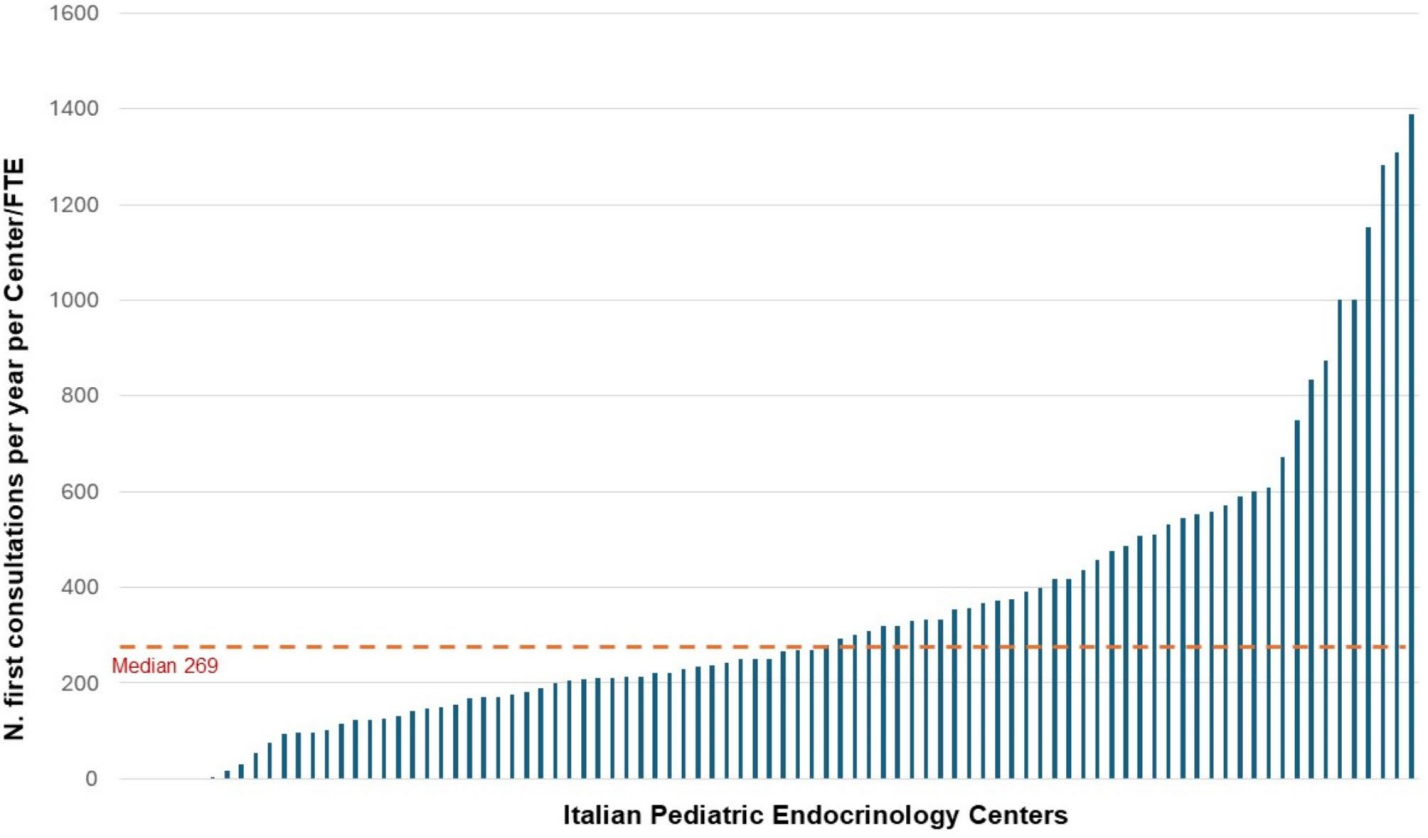
Total number of consultations reported *per* year *per* center [bars] for each medical FTE (missing data: 4/91 centers)

**Fig. 5:**
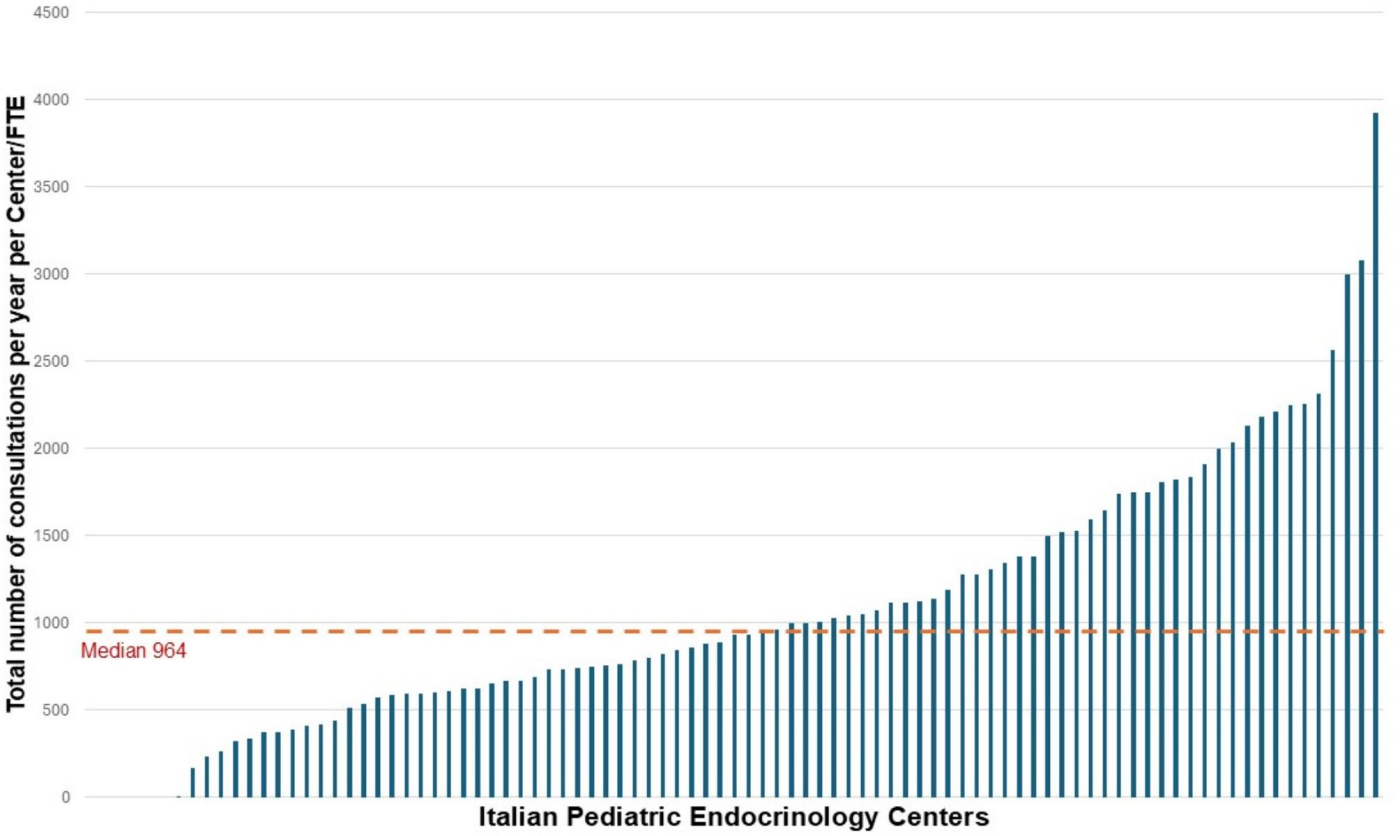
Number of first consultations per year per center [bars] for each medical FTE (missing data: 4/91 centers)

A mean of 3,891 patients was followed-up *per* region (median 3212, IQR 3732). Overall, a mean of 1148 consultations *per* year per center was reported for each medical FTE. In detail, a mean of 367 first consultations (Figs. 4) and 786 follow-up consultations *per* year *per* centre for each medical FTE was reported (Fig. 5).

### Rare diseases

The prevalence of rare diseases managed by each centre was also investigated. An average of 110 patients were followed *per* year *per* center (median value 30 patients). In this regard, non-homogeneous results were reported, with four centers taking care of 3810 patients with rare diseases out of 9876 patients. These were also the centers with the highest medical FTEs, thus, with more personnel, and located in large towns.

Overall patients with rare diseases represented 14.2% of the total patients followed-up.

### Hormonal stimulation tests

Stimulation tests were performed in 86/91 centers (94.5%). An average of 240 stimulation tests *per* year *per* center was reported.

Different types of dynamic tests were described including Gonadotropin-releasing hormone (GnRH), standard, low-dose and short Synacthen tests, arginine, growth hormone releasing hormone (GHRH), GHRH plus arginine, glucagon, and clonidine stimulation tests, prolactin curve, oral glucose tolerance, and insulin tolerance tests.

### Healthcare providers

The total numbers of HCPs employed in the 91 centers was analyzed. Pediatric endocrinologists were 271 (36 / 271 being academics). Fourteen centers had adult endocrinologists working in the team. Among the medical doctors, 27 were trainees, fellows, or PhD students.

In addition, 44 professional figures were categorized as “others” including psychologists, biologists, social workers, dieticians, nutritionists, secretaries, psychiatrists, and physiatrists.

Ninety-four pediatric endocrinologists had temporary contracts and 265 permanent contracts. Several employees were working in more than one section in their units, and, thus, only some of their time was dedicated to pediatric endocrinology.

### Full time equivalent calculations for the personnel

The effective time spent in pediatric endocrinology for each category of HCPs is reported in Table 1.

Overall, a mean FTE value of 0.56 for physicians, 0.49 for nurses, 0.31 for dieticians, and 0.13 for psychologists was found. Regarding the HCPs with a permanent contract, the average FTE was 0.56 for pediatric endocrinologists, 0.61 for nurses, 0.47 for dietitians, and 0.44 for psychologists (Table 1).

**Table 1:** Effective time spent in paediatric endocrinology for each category of the healthcare provider using the full time equivalent (FTE) as reference

HCPs [centres *N*. 91]	Total FTE	Mean FTE
Medical doctors [n. 271]	107,88	0.56
Nurses [n. 265]	78.84	0.61
Dietitians [n. 70]	24.05	0.47
Psychologists [n.41]	12.30	0.44

Based on the ideal need of HCPs for pediatric patients with diabetes established by ISPAD [12], considering the average number of rare diseases followed up per center (N.110), the suggested number of FTE pediatric endocrinologists should be 0.75-1, 1-1.25 for nurses, 0.5 for dietitians, and 0.3 for psychologists, respectively (Table 2).

Considering the medical need in terms of FTE, the missing number of HCPs according to this ideal standard of care was calculated (Table 2). We found that 80% of needed pediatric endocrinologists, 89% of required nurses, 93% of required dietitians, and 94% of the needed psychologists are currently lacking (Table 2).

**Table 2:** Suggested number for each category of healthcare providers for 100 rare disease patients, and calculation of missing healthcare providers

Healthcare providers	Suggested number * [*N*. for 100 patients]	Medical need [FTE-*N*. minimum]	Healthcare providers [FTE -*N*.]	Missing health providers [FTE *N*.]	Missing health providers [%]
Medical doctors	0.75-1	525	108	417	80
Nurses	1.0-1.25	700	79	621	89
Dietitians	0.5	350	24	326	93
Psychologists	0.3	210	12	198	94

* Suggested healthcare providers minimum number every 100 patients. ISPAD criteria [12.]

In addition, a lack of nurses (10.6% of the centers), dietitians (44.6% of the centers), and psychologists (55.3% of the centers) was also reported from the centers based on their historical organic staffing.

Based on the survey responses, 20 pediatric endocrinologists will be retiring within the next two years. Finally, administrative personnel as a support is lacking throughout the country and its function is taken up by nurses and medical doctors.

### Education and treatment for growth hormone deficiency

Education and training for the administration of growth hormone (GH) with specific devices was provided by a variety of HCPs in the different centers.

In 22/91 centers, training was provided by medical doctors, while in 15/91 by company specialists, in 33 by both doctors and specialists, and in 18 centers all the staff was involved in patient education. Finally, 3/91 centers did not prescribe GH treatment.

### Education for treatments and/or use of specific related devices for specific conditions other than growth hormone deficiency

How medications, including rare diseases, were prescribed, administered, and the mode of education/training was also evaluated (e.g. burosumab, vosoritide, mecasermin, liraglutide, steroids, gonadotropins, GnRH agonists).

In 34/91 centres, doctors were responsible for the patients’ education, in 15/91 company specialists, and in 14 both doctors and specialists. All the staff was involved in patients’ education in 18 centers, while 10 centers did not prescribe any of these therapies.

Data concerning nurses were unreported, probably due also to the shortage and because having different expertise in other fields.

## Discussion

This comprehensive overview of pediatric endocrinology activity in Italy highlights on the one hand a good distribution of centers throughout Italy, on the other an unequal distribution of these reflecting possibly unequal access to services for the population: Molise in 2021 had no centers whereas Lombardy, Piedmont, Emilia-Romagna, and Puglia had from 7 to 11 centers, being Lombardy, Piedmont and Emilia-Romagna the regions with the largest populations at variance with Puglia [9]. Worthy of note, these three regions also had the greater number of patients followed-up and of medical personnel at variance with Puglia which has a smaller population and more dedicated medical personnel to paediatric endocrinology.

Moreover, it should be noted that Lazio, the second Italian most populous region, was among the regions with the highest number of medical personnel but the total number of patients followed-up was lower compared to that of Lombardy, Piedmont, and Emilia-Romagna. Of note, Campania, despite being the third most populous region, and having six ISPED centers, had fewer medical personnel and total number of patients followed-up than the aforementioned three regions. More specifically, the number of patients followed-up in Campania was almost half of that of less populous regions such as Piedmont or Emilia Romagna. This might reflect different regional policies potentially encouraging both migration of patients and personnel. However, we cannot exclude a bias related with the fact that we censed centers having medical doctors being ISPED members. Notwithstanding, as there are no other pediatric endocrine societies in Italy, we assume we are not far from truth. Of note, the fact that only 6 centers in Italy were independent units, whereas all other centers were embedded in other units of pediatrics reflecting possibly a limited interest in this pediatric subspecialty by hospitals and institutions.

Inequalities in pediatric endocrine practice have been reported both in developing countries and other states of the developed world [14–16]. For this reason, harmonization of this imbalance has been recognized as an urgent need [14, 15]. To do this, European standard requirements for training institutions, trainers, and trainees have been released and are available [7].

Considering the shortage of pediatricians in Italy, we were surprised that 94/271 pediatric endocrinologists did not have a permanent position. This could be possibly due to scarcity of funds just after the COVID pandemic, insufficient to make permanent positions, however, the causes are difficult to tell. Definitely, the survey highlighted on average a very significant work burden for pediatric endocrinologists, and rare diseases account for a significant part of the work, and most are complex chronic conditions requiring a high level of expertise. Both rare and chronic diseases will be increasing with years along with the improved diagnostic abilities and new treatments arriving on the market. Rare endocrine diseases have been estimated to be more than 440 [17–19] and in most cases chronic and life-threatening [19, 20]. Although there is a lack of robust pediatric epidemiological data in the field, approximately 70% of rare endocrine diseases in children are lifetime and disabling conditions requiring a multidisciplinary approach [18, 20–22]. Therefore, despite the development of strategic plans (including financial and educational resources), the large spectrum of clinical unmet needs experienced by patients with rare diseases still represents a great challenge for experts [18, 20].

The lack of personnel we registered and calculated for Italy was striking and brings worries for the future of this essential pediatric subspecialty. Interestingly, in the United States a trend estimating a shortage of 100 up to 200 pediatric full-time endocrinologists from 2014 to 2025 [13, 23, 24] was highlighted, and a recent analysis of the Pediatric Endocrine Society Task Force reported alarming data on the future of the pediatric endocrinology as the lack of trained subspecialists and the increased number of patients with endocrine conditions [25, 26]. These numbers are, however, below those calculated for Italy. Moreover, in the United States, the increased number of active pediatric endocrinologists from 2011 to 2018, data reported that 21% of these were aged > 60 years and a further considerable percentage was involved in academic rather than clinical practice [25], suggesting a potential underestimation of the effective clinical endocrine needs [25]. In Italy, only 36/91 centers reported to be academical clinical practices. The data evidenced also that most pediatricians were only partially dedicated to pediatric endocrinology whereas the numbers would suggest we need a much larger number of pediatric endocrinologists.

Therefore, ensuring an optimal capacity of the workforce for pediatric endocrinology represents one of the most important current challenges, and this together with the underlined shortage of these subspecialists. In addition, the prevalence of pediatric endocrine disorders is increasing worldwide [27, 28] along with the lack of existing centers for transition to adult care, thus, a further clinical overload for pediatric endocrinologists has been foreseen [25].

A further matter of concern is represented by the fact that recognition of pediatric endocrinology as a subspecialty still varies across European countries [2, 3], including states with fully approved pediatric endocrinology as subspecialty (e.g. United Kingdom), with built-up training programs but no approved subspecialty (e.g. Italy, France, and Germany), or with no subspecialty (mainly Eastern countries) [2]. Commonly, clinical settings for this subspecialty are represented by university centers and dedicated clinical services in government-funded hospitals staffed by trained specialists [13]. Pediatric endocrinology has become a recognized subspecialty especially in European Western countries over the past 60 years [5, 29]. However, taking into account the above considerations, it is not a surprise that children with endocrine disorders are still often referred to adult endocrinologists because of a substantial clinical gap in pediatric endocrine care [29]. For these reasons, scientific societies such as the ESPE and the Lawson Wilkins Pediatric Endocrine Society in the United States have made great efforts to provide a better definition of educational standards and training programs in the field, to improve and harmonize the quality of care of pediatric endocrine patients among different countries [7, 29, 30]. In particular, it is suggested that training in European countries should include a mandatory 4-year period in general pediatric training followed by a 2–3-year clinical training in pediatric endocrinology [5, 7]. Acquisition of research expertise through a formal period of clinical or laboratory research is also recommended [7].

To potentially overcome this lack of specialists, a wider exposure of medical students to the field and of residents to experiences in endocrinology have been recommended to enhance career interest in this subspecialty [24]. In addition to the definition of knowledge and skills standards required for endocrine practice at the tertiary care level, the overall improvement of the quality of care through the development of powerful regional networks and of staff with specific expertise (including nursing, medical, and technical personnel) has been also considered by experts for this purpose [5, 7]. Development of nursing expertise has been also highly valued [5]. Indeed, nursing responsibilities are extremely different across continents, ranging from general pediatric nursing care, growth assessment, dynamic hormonal tests execution to medication prescription [5, 20].

## Conclusions

At present pediatric endocrinology shows a significant burden of activity with severe shortage of personnel. The data from this survey clearly highlighted that the lack of nurses in Italy is even greater than that of medical doctors not to mention other HCPs.

To the best of our knowledge, this study represents the first analysis of pediatric endocrinology workforce in Italy. In consideration of the complexity of endocrine disorders management, addressing the alarming shortage of trained personnel should represent a priority for institutions as they must guarantee an optimal clinical care of pediatric endocrine patients.

Recruitment of future subspecialists and official recognition of paediatric subspecialties should be of paramount importance and addressed by policy makers in order to develop strategic programs to ensure optimal care. Recognizing pediatric endocrinology as a subspecialty and offering appropriate training programs would represent a significant step further.

## Electronic supplementary material

Below is the link to the electronic supplementary material.

Supplementary Material 1

## Data Availability

Data are available from the corresponding author upon reasonable request.

## Abbreviations

ISPED: Italian Society for Pediatric Endocrinology and Diabetology
ESPE: European Society of Paediatric Endocrinology
FTE: Full-time equivalent
GH: Growth Hormone
GHRH: Growth hormone releasing hormone
GnRH: Gonadotropin Releasing Hormone

## References

[1] Kiess W, Penke M, Kratzsch J, Vogel M, Kapellen T, Hoppmann J, et al. Pediatric endocrinology is pediatrics is public health. J Pediatr Endocrinol Metab. 2017;304:371–4. 10.1515/jpem-2017-0109 28358715

[2] Lebl J, Luczay A, Darendeliler F, Verkauskiene R. Paediatric endocrinology Subspecialty - The European map, 55 years later. Horm Res Paediatr. 2018;89(1):71–2. 29402866 10.1159/000481505

[3] ESPE Position Statement for Paediatric Endocrinology Subspecialty. Horm Res Paediatr. 2016;861:1–2. 10.1159/000447502 27379931

[4] Bhutta ZA, Norris SA, Roberts M, Singhal A. The global challenge of childhood obesity and its consequences: what can be done? Lancet Glob Health. 2023;118:e1172–3. 10.1016/S2214-109X(23)00284-X 37474221

[5] Savage MO, Donaldson MDC, Davies JH, Storr HL. Key stages in the development and establishment of paediatric endocrinology: A template for future progress. Horm Res Paediatr. 2024;97[1]:22– 7. 10.1159/000530841 PMC10836736 37166328

[6] http://attiministeriali.miur.it/media/248802/allegato_dm_68.pdf

[7] Busiah K, Peet A, Tornese G, Weintrob N, Schulga J, Hamza RT et al. The 2021 European training requirements in paediatric endocrinology and diabetes. Horm Res Paediatr. 2021;94[11–12]:441–7. 10.1159/000520073 34638127

[8] European Training Requirements for Competency in Paediatric Endocrinology and Diabetes. Syllabus - https://www.eurospe.org/wp-content/uploads/2022/12/etr-ped-endocrinology-espe-approved-24april2021-final-1-1.pdf

[9] https://www.istat.it/it/archivio/295834 Last accessed 23rd June 2024.

[10] https://ec.europa.eu/eurostat/statistics-explained/index.php?title=Glossary:Full-time_equivalent_[FTE]. Last accessed 23rd May2024.

[11] https://www.indeed.com/hire/c/info/full-time equivalent#:~:text = The%20full%2Dtime%20equivalent%20or,employees%20would%20be%201.0%20FTE Accessed 4th April 2024.

[12] Pihoker C, Forsander G, Fantahun B, Virmani A, Corathers S, Benitez-Aguirre P, et al. ISPAD clinical practice consensus guidelines 2018: the delivery of ambulatory diabetes care to children and adolescents with diabetes. Pediatr Diabetes. 2018;19(Suppl 27):84–104. 30144259 10.1111/pedi.12757

[13] Vigersky RA, Fish L, Hogan P, Stewart A, Kutler S, Ladenson PW, et al. The clinical endocrinology workforce: current status and future projections of supply and demand. J Clin Endocrinol Metab. 2014;999:3112–21. 10.1210/jc.2014-2257 24940655

[14] Savage MO, Cassorla FG, Gluckman PD, Grueters-Kieslich A, Raghupathy P, Silink M, et al. Global inequalities in paediatric endocrine practice: statement of minimal acceptable care. Statement from the international societies for paediatric endocrinology. Horm Res. 2006;653:111–3. 10.1159/000091278 16462146

[15] Odundo GO, Ngwiri T, Otuoma O, Chanzu NM. Developing equity in capacity of paediatric endocrinology subspecialists worldwide. Lancet Diabetes Endocrinol. 2016;43:204–5. 10.1016/S2213-8587(16)00035-8 26827113

[16] Zacharin M, Chanoine JP, Cassorla F, Brink S, Hanas R, Fideleff HL et al. Promoting excellence in the care of pediatric endocrine diseases in the developing world. Pediatrics. 2013;131[2]:e573-8. 10.1542/peds.2012-0848 23339226

[17] Chung CCY, Hong Kong Genome P, Chu ATW, Chung BHY. Rare disease emerging as a global public health priority. Front Public Health. 2022;10:1028545. 36339196 10.3389/fpubh.2022.1028545 PMC9632971

[18] Reincke M. A Hokken-Koelega Perspectives of the European society of endocrinology [ESE] and the European society of paediatric endocrinology [ESPE] on rare endocrine disease. Endocrine 2021;71(3):539–41. 33740222 10.1007/s12020-021-02652-x PMC7976677

[19] The Lancet Diabetes E. Spotlight on rare diseases. Lancet Diabetes Endocrinol. 2019;7[2]:75. 10.1016/S2213-8587(19)30006-3 30683214

[20] Tumiene B, Peters H, Melegh B, Peterlin B, Utkus A, Fatkulina N, et al. Rare disease education in Europe and beyond: time to act. Orphanet J Rare Dis. 2022;17(1):441. 36536417 10.1186/s13023-022-02527-y PMC9761619

[21] Davies K, Collin J. Understanding clinical investigations in children’s endocrinology. Nurs Child Young People. 2015;278:26–36. quiz 7. 10.7748/ncyp.27.8.26.s24 26448126

[22] Austin CP, Cutillo CM, Lau LPL, Jonker AH, Rath A, Julkowska D et al. Future of rare diseases research 2017–2027: an IRDiRC perspective. Clin Transl Sci. 2018;11[1]:21– 7. 10.1111/cts.12500 PMC5759721 28796445

[23] Rizza RA, Vigersky RA, Rodbard HW, Ladenson PW, Young WF Jr., Surks MI et al. A model to determine workforce needs for endocrinologists in the United States until 2020. J Clin Endocrinol Metab. 2003;88[5]:1979-87. 10.1210/jc.2002-021288 12727941

[24] Tsai K, Long C, Liang TZ, Napolitano J, Khawaja R, Leung AM. Driving factors to pursue endocrinology training fellowship: empirical survey data and future strategies. J Clin Endocrinol Metab. 2022;107(6):e2459–63. 35165741 10.1210/clinem/dgac087

[25] Allen DB, Aye T, Boney CM, Eugster EA, Misra M, Singer K, et al. Sustaining the pediatric endocrinology workforce: recommendations from the pediatric endocrine society workforce task force. J Pediatr. 2021;233:4–7. 33137317 10.1016/j.jpeds.2020.10.063

[26] Freed GL, Boyer DM, Van KD, Macy ML, McCormick J, Leslie LK. Variation in Part-Time work among pediatric subspecialties. J Pediatr. 2018;195:263–8. 29395185 10.1016/j.jpeds.2017.11.060

[27] Ocal G. Current concepts in disorders of sexual development. J Clin Res Pediatr Endocrinol. 2011;3(3):105–14. 21911322 10.4274/jcrpe.v3i3.22 PMC3184510

[28] Hoe FM, Darbinian JA, Greenspan LC, Lo JC. Hemoglobin A1c and type 2 diabetes incidence among adolescents with overweight and obesity. JAMA Netw Open. 2024;7[1]:e2351322. 10.1001/jamanetworkopen.2023.51322 PMC10794942 38231515

[29] Illig R, Laron Z, Visser HK. From the paediatric endocrinology club to the European society for paediatric endocrinology: the early years of ESPE. Pediatr Endocrinol Rev. 2011;9[1]:417– 21. 22783639

[30] Oberfield SE, Rogol AD, Miller WL. A brief history of the pediatric endocrine society [PES]. Horm Res Paediatr. 2022;956:510–4. 10.1159/000526439 36446318

