# Supplementary material for: Landscape of paediatric endocrine clinical practice in Italy: results from a survey of the Italian Society for Paediatric Endocrinology and Diabetology (ISPED)
Source: Ital J Pediatr. 2025 Mar 24;51:90. doi: 10.1186/s13052-025-01940-w (PMC11934765; doi:10.1186/s13052-025-01940-w)
Supplement: Supplementary file 1 — Supplementary Material 1 [file 13052_2025_1940_MOESM1_ESM.docx]

**PAEDIATRIC ENDOCRINOLOGY ACTIVITY IN ITALY : A SURVEY OF THE ISPED CENTERS IN ITALY**

DETAILS OF THE HEAD OF THE CENTER

Surname:

Name:

Email address:

Phone number:

Role of the Center Head:

INFORMATION ON THE CENTER

Name:

City:

Province:

Region:

Phone number:

Typology:

- clinic within a unit

– division within a unit

– division within a department

– independent unit

Position:

- clinic in a local community health facility

– hospital

– academic center embedded in a teaching hospital

INFORMATION ON PERMANENT STAFF

Number of clinical doctors:

Number of academic doctors:

Are doctors all pediatricians? Yes – No

Are there any adult endocrinologists? Yes – No;

If YES give Number:

Number of trainees/residents in Paediatrics, fellows, or PhD students:

Full Time Equivalent [(38 – hours per week spent not in endocrinology) / 38] of every doctor:

Number of nurses:

Full Time Equivalent [(38 – hours per week spent not in endocrinology) / 38] of every nurse:

Number of dietians:

Full Time Equivalent [(38 – hours per week spent not in endocrinology) / 38] of every dietician:

Number of psychologists:

Full Time Equivalent [(38 – hours per week spent not in endocrinology) / 38] of every psychologist:

Number of other staff members/operators:

Full Time Equivalent [(38 – hours per week spent not in endocrinology) / 38] of every operator:

INFORMATION ON TEMPORARY STAFF

Number of doctors:

Number of nurses:

Number of dietitians:

Number of psychologists:

Number of other health staff/ operators:

1. doctors who will be missing in the next two years:
2. Ddoctors who will retire in the next two years:

Other staff who will be missing in the next two years:

Other staff who will retire in the next two years:

PATIENTS AND SERVICES

Patient followed- age range (yr):

Total number of patients seen in 2021 at the center:

Number of patients with rare diseases:

Number of non-rare syndromes and chromosopathies:

Total number of first visits (year 2021):

Total number of follow-up visits (year 2021):

How many days per week does the Center carry out its clinical activities?

Is there a telephone service available for patients? No

– Landline phone service at set time

- Landline or mobile phone service 24 h as an unofficial service

- Landline or mobile telephone service five days a week

Are stimulation tests performed in the Center?

Total number of tests (year 2021):

Type of tests:

Who provides education and training on GH devices?

Medical doctors

– Nurses

– All the staff

– Company specialists

Who provides education and training on therapy with IGF-I/burosumab/other medications for rare diseases? Doctors

– Nurses

– All the staff

– Company specialists

Does the Center incorporate a Pediatric Diabetology service?

IS there an independent Pediatric Diabetology Center?
